# Blood and cerebrospinal fluid biomarker changes in patients with HIV-associated neurocognitive impairment treated with lithium: analysis from a randomised placebo-controlled trial

**DOI:** 10.1007/s13365-023-01116-4

**Published:** 2023-02-15

**Authors:** Lindokuhle Thela, Eric Decloedt, Henrik Zetterberg, Magnus Gisslén, Maia Lesosky, Melanie Gleich, Eleni Koutsilieri, Carsten Scheller, Abdul Hye, John Joska

**Affiliations:** 1 HIV Mental Health Research Unit, Department of Psychiatry and Mental Health, Neuroscience Institute, University of Cape Town, E Floor, Neuroscience Centre, Anzio Road, Groote Schuur Hospital, Observatory, 7925 Cape Town, South Africa; 2 Division of Clinical Pharmacology, Department of Medicine, Stellenbosch University, Cape Town, South Africa; 3 Department of Psychiatry and Neurochemistry, Institute of Neuroscience and Physiology, the Sahlgrenska Academy at the University of Gothenburg, Mölndal, Sweden; 4 Clinical Neurochemistry Laboratory, Sahlgrenska University Hospital, Mölndal, Sweden; 5 Department of Neurodegenerative Disease, UCL Institute of Neurology, Queen Square, London, UK; 6 UK Dementia Research Institute at UCL, London, UK; 7 Hong Kong Center for Neurodegenerative Diseases, Clear Water Bay, Hong Kong, China; 8 Department of Infectious Disease, Institute of Biomedicine, the Sahlngreska Academy at the University of Gothenburg, Gothenburg, Sweden; 9 Department of Infectious Disease, Sahlgrenska University Hospital, Region Västra Götaland, Gothenburg, Sweden; 10 Division of Epidemiology and Biostatistics, School of Public Health and Family Medicine, University of Cape Town, Cape Town, South Africa; 11 Institute of Virology and Immunobiology, University of Würzburg, Würzburg, Germany; 12 King’s College London, Institute of Psychiatry, Psychology and Neuroscience, and NIHR Biomedical Research Centre for Mental Health and Biomedical Research Unit for Dementia at South London and Maudsley NHS Foundation, London, UK

**Keywords:** HIV, HAND, Biomarkers, Lithium

## Abstract

HIV-associated neurocognitive disorders (HAND) persist in the era of antiretroviral therapy (ART). Thus, ART does not completely halt or reverse the pathological processes behind HAND. Adjuvant mitigating treatments are, therefore, prudent. Lithium treatment is known to promote neuronal brain–derived neurotrophic factors (BDNF). Lithium is also an inhibitor of glycogen synthase kinase-3 beta (GSK-3-β). We analyzed biomarkers obtained from participants in a randomized placebo-controlled trial of lithium in ART-treated individuals with moderate or severe HAND. We assayed markers at baseline and 24 weeks across several pathways hypothesized to be affected by HIV, inflammation, or degeneration. Investigated biomarkers included dopamine, BDNF, neurofilament light chain, and CD8 + lymphocyte activation (CD38 + HLADR +). Alzheimer’s Disease (AD) biomarkers included soluble amyloid precursor protein alpha and beta (sAPPα/β), Aβ38, 40, 42, and ten other biomarkers validated as predictors of mild cognitive impairment and progression in previous studies. These include apolipoprotein C3, pre-albumin, α1-acid glycoprotein, α1-antitrypsin, PEDF, CC4, ICAM-1, RANTES, clusterin, and cystatin c. We recruited 61 participants (placebo = 31; lithium = 30). The age baseline mean was 40 (± 8.35) years and the median CD4 + T-cell count was 498 (IQR: 389–651) cells/μL. Biomarker concentrations between groups did not differ at baseline. However, both groups’ blood dopamine levels decreased significantly after 24 weeks (adj. *p* < 002). No other marker was significantly different between groups, and we concluded that lithium did not confer neuroprotection following 24 weeks of treatment. However, the study was limited in duration and sample size.

**Supplementary Information:**

The online version contains supplementary material available at 10.1007/s13365-023-01116-4.

## Introduction

Since the advent of antiretroviral therapy (ART), HIV management has improved dramatically, resulting in a decrease in early mortality in people with HIV (PWH) (Trickey et al. 2017). Despite this, HIV-associated neurocognitive disorders (HAND) continue to affect a significant proportion of PWH who are adequately treated (Heaton et al. 2010). Furthermore, HAND is an independent prognostic marker of mortality (Naveed et al. 2021). Globally 44.9% of PWH meet the Frascati criteria for HAND (Wei et al. 2020). Asymptomatic neurocognitive impairment (ANI) accounts for 26.5% of this number, whereas mild neurocognitive disorder contributes 8.5%, and HIV-associated dementia contributes 2.1%. Though ANI has no clinical significance, the presence of ANI is associated with a 4–sixfold risk of developing symptomatic HAND (Grant et al. 2014).

The high reported rates of ANI in PWH are, however, controversial. There is growing concern that relying solely on neuropsychological performance can lead to false positives of up to 20% (Gisslen et al. 2011). Further, many sociodemographic factors may account for low cognitive performance in PWH. This may lead to an overestimation of the prevalence of ANI. Some PWH with ANI may also perform within a spectrum of normality during neuropsychological assessments (Nightingale et al. 2021). Accordingly, to validate ANI, biomarkers should be developed to distinguish individuals with subtle neuropathology in the brain from those who perform within normal limits.

The pathophysiology of HAND is complex and multifactorial. The viral pathway involves neuronal dysfunction and irreversible neuronal injury, which often correlates with the level of virus circulating in the plasma (Marcotte et al. 2003). The presence of HAND during adequate viral suppression could result from pre-treatment injury called legacy effect (Qu et al. 2022) and persistent viral replication in the CNS compartment, as in the case of cerebrospinal fluid (CSF) viral scape (Nightingale et al. 2014). Chronic compartmentalized neuroinflammation persists even during viral suppression (Ulfhammer et al. 2018). Eden et al. (2016) found that during adequate ART treatment, neopterin levels (a marker of CNS immunoactivity) are higher in those with ANI compared to those with normal cognition (Eden et al. 2016). Similarly, a study by Yuan et al. (2013) also found a strong correlation between HAND and CSF inflammation during adequate viral suppression (Yuan et al. 2013).

Preliminary results from observational and experimental studies suggest that this form of chronic neuronal injury may set off neurodegeneration. In addition, there has been a steady rise in the number of PWH living beyond the age of 50 years (Autenrieth et al. 2018). Therefore, identifying potential neuroprotective compounds or therapies has become even more pressing. An ideal therapy should rectify the pathways implicated in the pathophysiology of HAND (Lindl et al. 2010; Rumbaugh et al. 2008; Turchan et al. 2003). Drugs tested in previous clinical trials include memantine, minocycline and paroxetine (Sacktor et al. 2017; Simioni et al. 2013; Schifitto et al. 2007). Two smaller pilot trials investigated the efficacy of lithium in HAND, finding small imaging and clinical evidence of effect (Decloedt et al. 2016; Schifitto et al. 2009). Lithium is a well-established treatment for bipolar mood disorders (Malhi and Outhred 2016). Besides the ability to treat and prevent relapses in bipolar mood disorders, lithium can protect neurons from inflammation and neurotoxicity. Lithium is known to work by inhibiting the GSK-3-β and regulates neurotransmitters such as dopamine and glutamate. Lithium also promotes the expression of BDNF in the neurons.

GSK-3-β is a serine protein kinase that promotes cellular apoptosis (Thornton et al. 2017). Increased expression of GSK-3-β in the CNS is associated with neurodegeneration in disorders such as AD. GSK-3-β induces DRP1-Ser616 phosphorylation, resulting in a dysfunctional mitochondrial alternation in fission. In an experiment with mouse neurons, GSK-3-β hyperactivity resulted in neuronal cell death in some areas of the hippocampus and the cortex (Thornton et al. 2017). There is an increase in CNS tissue GSK-3-β activity during HIV infection. Maggirwar et al. (1999) experiment on the activity of rat neurons GSK-3-β after exposure to Tat showed that Tat was associated with an enhanced expression of GSK-3-β, resulting in neurotoxicity (Maggirwar et al. 1999). Treating the neurons with lithium caused a reduction in the GSK-3-β and the attenuation of neurotoxicity (Maggirwar et al. 1999). GSK-3-β is a promotor of neuroinflammation, another primary pathway of neuronal injury in patients with HAND.

Various surrogate biomarkers can be analyzed from plasma and CSF to assess the effectiveness of treatments against HAND. Some of the biomarkers have been studied extensively in HAND and found to be highly sensitive and specific for neuronal injuries, such as the neurofilament light chains (NfL) (Gisslén et al. 2016). Blood and CSF concentrations of NfL positively correlate with the severity of the neuronal injury and cognitive impairment in PWH (Gisslén et al. 2016; Anderson et al. 2018). Low plasma and CSF BDNF are associated with poor cognitive function (Levada et al. 2016). HIV decreases BDNF levels by preventing the conversion of passive proBDNF into mature BDNF (Bachis et al. 2012). Peripheral (blood) and central dopamine (DA) pathways are both involved in the pathogenesis and severity of HAND. Peripheral DA is linked with a dose-dependent entry of HIV into the macrophages, which facilitates HIV neuro-invasion (Gaskill et al. 2014). There is a correlation between a reduction in central DA levels and the severity of neurocognitive impairment in PWH. In PWH, an increase in the expression of human leukocyte antigen (HLA) DR and CD38 + on CD8 + T-lymphocytes (HLA-DR + CD38 + CD8) is associated with the continuing neuronal injury and progression of HAND (Liu et al. 1997; Robertson et al. 2020; Ratto-Kim et al. 2018).

Because of similarities between AD and HAD, such as the chronic inflammatory state, research has been conducted on CSF amyloid and tau protein metabolism in patients with HAND. Aβ plaque synthesis may be triggered by chronic inflammation, microglial activation, and disruption of the blood–brain barrier (BBB) (Noe et al. 2020), which is also seen in HIV. Furthermore, in vitro studies have found that HIV viral protein Tat has a high affinity for the surface of Aβ fibrils (Hategan et al. 2017). Tat interacts with this surface, promoting Aβ plaques synthesis (Hategan et al. 2017). HIV-infected patients also have a higher incidence of Aβ deposits in the brain (Green et al. 2005; Esiri et al. 1998). Clifford et al. (2009) found reduced CSF Aβ_1-42_ in PWH diagnosed with HAND compared to healthy matched controls. The concentration of CSF A_β1-42_ was not different to that of patients with mild Alzheimer’s type dementia (Clifford et al. 2009). However, CSF tau concentration was higher in the Alzheimer’s type dementia participants when compared to that of the control and HAND participants.

Similarly, in a study by Gisslen et al. (2009), the CSF Aβ_1-42_ was lower in participants with AIDS dementia complex (ADC) compared to cognitively normal PWH (CNP) participants. While the high CSF tau protein metabolite was higher in ADC participants, it was not significantly different from that of the control and CNP participants (Gisslen et al. 2009). These findings suggest that HAND neuronal injury does not show the pattern of neuronal injury observed in AD. In recent years, several AD predictive biomarkers were validated as having high accuracy (87%), sensitivity (85%), and specificity (88%) in detecting the progression of mild cognitive impairment of the AD type (Hye et al. 2014). These markers are primarily inflammatory markers that are easily detectable in plasma. In addition to amyloid and tau, these markers may serve as potential surrogates for the HAND.

Lithium confers neuroprotection by modulating neurotransmission and preventing neurotoxicity (Malhi et al. 2013). In Neuro-HIV, viral proteins, namely Tat and gp120 interfere with dopamine and glutamate, resulting in neurotoxicity. For example, gp120 inhibits the dopamine transporter (DAT) reuptake of dopamine, causing prolonged postsynaptic neurostimulation. The result is the loss of neurons with a high density of dopamine receptors, such as in the basal ganglia. Many studies have shown that treatment with lithium enhances the expression of neuronal BDNF. BDNF promotes neuronal survival, growth, neuroplasticity, and learning (Quiroz et al. 2010). A reduction in the expression of neuronal BDNF occurs during HIV neuro-infection. The viral protein gp120 inhibits the conversion of proBDNF to BDNF by binding to the C–C chemokine receptor type 5, leading to a higher proBDNF/BDNF ratio which correlates with HAND severity.

In this study, we aimed to determine whether lithium can halt or reverse the injury caused by HIV in patients diagnosed with moderate to severe HAND who are on stable ART treatment. This study is the first to use biomarkers across several pathways hypothesized to be involved in HAND and AD.

## Methods

### Study design, participants, and setting

This study reports a secondary analysis of data collected prospectively during a 24-week randomized placebo-controlled clinical trial of lithium in patients with severe to moderate HAND who are on ART. This manuscript describes the changes observed in large blood and cerebrospinal fluid (CSF) biomarker data (Decloedt et al. 2016). According to the preliminary study, neither lithium nor placebo affected cognitive performance. However, in both treatment arms, there was an improvement in the global deficit score (GDS), likely attributable to the practice effect because of repeated neurocognitive testing.

### Methods of the parent study

Study participants were recruited from the Nolungile Site C clinic in Khayelitsha and followed up at Groote Schuur Hospital. The inclusion criteria were: ≥ 18 and ≤ 70 years, cognitive impairment defined by the GDS of ≥ 0.5, uninterrupted ART treatment for at least 6 months a plasma HIV RNA < 400 copies/ml. The exclusion criteria were participants taking lithium within 30 days of entering the study, acquired immune deficiency syndrome, history of substance use including benzodiazepines, presence of neurosyphilis, vitamin B12 deficiency, abnormal brain imaging results, and the presence of traumatic brain injury. In the end, the study recruited 66 patients. The participants were randomly assigned to a placebo arm = 34 (31 completed) and a lithium arm = 32 (30 completed). Demographic data and other outcome measures such as the GDS and blood and CSF biomarkers were collected during the first visit and at the end of the clinical trial. The clinical trial received ethical approval from the universities of Cape Town (071/2013) and Stellenbosch (M13/07/027). The registration number of the trial is PACTR201310000635418.

### Biomarker consideration

The biomarkers that were selected a priori were the fluid biomarkers that were the primary outcome objectives of the parent study. These biomarkers were blood CD8 + T lymphocyte activation and blood and CSF dopamine and BDNF. The post hoc biomarkers were the neurofilament light chain and Alzheimer’s disease–related biomarkers. All analytes (individual biomarkers or protein) were processed in single batches or one group using the same equipment, reagent, and technician.

### Statistical considerations

#### Sample size

We enrolled 54 participants for each treatment arm to account for about a 10% loss to attrition. The GDS has been shown to improve by a mean of approximately 0.13 in patients with mild to moderate (> 0.25 to < 0.75) and 0.6 in patients with severe HAND (> 0.75) in the population we studied (Joska et al. 2012). In a previous comparable study, it was found that 12 weeks of adjuvant lithium treatment in stable ART-treated patients improved GDS by a mean of approximately 0.3 (Letendre et al. 2006). We recruited participants with a similar profile to this study. However, our object was more conservative, and we aimed for a GDS difference of 0.25 (versus 0.3). For a power of 90% and alpha of 0.05, we needed a sample size of at least 49 participants per arm. GDS is an alternative method for determining cognitive impairment in HIV-positive individuals (Blackstone et al. 2012). The GDS was intended to be a user-friendly, automated approach highlighting performance deficits. The method considers the severity and the number of deficits in performance throughout the test battery while assigning less weight to performances within the normal range. GDS is preferred over the clinical rating scales when the aim is to identify severe levels of cognitive impairment.

#### Data analysis

The data were analyzed with GraphPad Prism 9. Categorical variables are presented as counts and or percentages. Continuous variables are presented as means (standard deviations) or medians (interquartile range), depending on the distribution. Bivariate and group comparisons were conducted with Chi-square or Fisher’s exact tests, or *t*-tests or Wilcoxon signed-rank tests, depending on the type and distribution of the variables. Paired tests were used when appropriate. A *p*-value of less than 0.05 was statistically significant. When there was statistical significance, we conducted a post hoc analysis using the Bonferroni test to correct for false discovery rate (FDR) because of multiple comparisons. Both arms were subjected to two analyses. First, at baseline and again at week 24, unpaired tests were used to compare biomarker levels between the placebo and lithium arms. Second, paired tests assessed each biomarker for longitudinal changes from before the intervention (week 0) to the end (24 weeks).

#### Blood CD8 + T lymphocyte activation measurement

Peripheral blood mononuclear cell (PBMC) was resuspended in PBS containing 0.5% BSA and stained with commercially available antibodies (CD8-FITC, CD38-PE, CD3-PerCPCy5, HLADR-APC, all from BD Biosciences) and analyzed by flow cytometry using a FACSCalibur flow cytometer (BD Biosciences). After gating the lymphocyte population in the FSC/SSC-plot, the following cell populations were defined: T-cells (CD3 +), CD8 + T-cells (CD3 + /CD8 +) and activated CD8 + T cells (CD3 + /CD8 + /CD38 + /HLADR +). PBMC were collected at baseline (week 0) and week 24 and stained with anti-CD3/CD8 to define CD8 + T cells and counterstained with anti-CD38/HLADR to detect the frequency of activated CD8 + T cells.

#### Plasma and CSF BDNF and DA concentrations

Plasma (all patients) and CSF (*n* = 35 at baseline and *n* = 18 at week 24) samples were collected at weeks 0 and 24 and subjected to BDNF enzyme-linked immunosorbent assay (ELISA). Plasma and CSF samples were inactivated at 56 °C for 30 min. BDNF (Abcam) and DA (Abnova) concentrations were determined by commercially available ELISA kits according to the manufacturers’ instructions. Briefly, for the BDNF ELISA, plasma samples were diluted at a ratio of 1:4 before ELISA, and CSF samples were analyzed undiluted. For the DA ELISA, CSF and plasma samples were analyzed undiluted. All ELISA DA experiments were run in one round on the same day using a single plate and reagents (one batch). Three experiments were conducted for the BDNF ELISA. All CSF BDNF ELISA experiments (before and post-intervention) were conducted in one batch. The plasma BDNF ELISA experiments were conducted in two batches at different points (before and post-intervention).

#### Plasma biomarkers of mild cognitive impairment to AD progression

The following plasma proteins were measured using multiplex bead assays (Luminex xMAP): acid glycoprotein (AGP), apolipoprotein C3 (ApoC3), pre-albumin, alpha-1 antitrypsin (A1AT), pigment epithelium-derived factor (PEDF), complement component 4 (CC4), intercellular adhesion molecules-1 (ICAM-1), regulated on activation, normal T cell expressed and secreted (RANTES), clusterin and cystatin c. Median fluorescent intensity (MFI) of the xMAP assays were measured using the xPONENT 3.1 (Luminex Corporation) The MFI were exported into Sigma Plot (Systat Software; Version 12.5) for estimation of protein concentrations using a 5-parameter logistic fit.

#### Plasma and CSF neurofilament light chains

CSF NfL concentration was measured using a commercially available sandwich ELISA according to the kit manufacturer’s instructions (UmanDiagnostics, Umeå, Sweden). Plasma NfL concentration was measured using the commercially available Single molecule array (Simoa) NF-Light assay (Quanterix, Billerica, MA, USA), according to a protocol previously described in detail (Gisslén et al. 2016). All samples were batched and analysed in the same run.

#### CSF amyloid proteins

sAPPα/β concentrations were measured in CSF with a duplex immunoassay and electrochemiluminescence detection (Meso Scale Discovery, Rockville, MD, USA). CSF Aβ38, Aβ40, and Aβ42 concentrations were measured using a triplex immunoassay with electrochemiluminescence detection (Meso Scale Discovery, Rockville, MD, USA). The measurements were performed in one round of experiments using one batch of reagents by board-certified laboratory technicians blinded to clinical data. Intra-assay coefficients of variation were below 10%.

#### The study protocol and approval

University of Cape Town Faculty of Health Sciences Human Research Ethics Committee approved this study (HREC REF: 772/2020).

## Results

### Participants’ baseline characteristics before randomisation

This study was conducted over 18 months (December 2013–June 2015). Sixty-six participants were enrolled (placebo arm = 34 and lithium arm = 32), and 61 completed the study (placebo arm = 31 and lithium arm = 30). All participants were black Xhosa-speaking Africans**.** The participants in the two treatment arms did not differ in terms of age (*p* = 0.48), gender (*p* = 0.26), years of education (*p* = 0.81), CD4 + T lymphocyte counts (*p* = 0.80), GDS (*p* = 0.79) or time on ART (*p* = 0.64) (Table 1).

**Table 1:** Characteristics of participants at enrolment

**Characteristic**	**Treatment groups**		
	**Placebo *n* = 34**	**Lithium *n* = 32**	***p*-value**
^a^Age (mean ± SD) years	40.53** ± **8.71	39.03** ± **8.09	0.48
Race			
Black African	100%	100%	
^b^Gender			
Female	28 (82%)	30 (94%)	0.26
Males	6 (18%)	2 (6%)	
^c^Years of education			
< 10 years	16 (47%)	14 (44%)	
≥ 10 years	18 (53%)	18 (56%)	0.81
^c^CD4 count: median (IQR)	498 (379 – 665)	502 (391 – 649)	0.8
^c^GDS: median (IQR)	1.12 (0.82 – 1.53)	1.10 (0.8 – 1.5)	0.79
Antiretroviral therapy			
NNRTI-based	30(88%)	26(81%)	0.33
PI-based	4(12%)	6(19%)	
^c^Time on treatment(months): median (IQR)	40 (25 – 73)	51 (22 – 77)	0.64

*SD* Standard deviation, *IQR* Interquartile range, *NNRTI* Non-nucleoside reverse transcriptase inhibitors, *PI* Protease inhibitors

^a^Unpaired *t*-test

^b^Fischer exact test

^c^Mann-Whitney test

### Blood biomarkers

No differences were observed between the two arms concerning individual biomarker concentrations. Biomarkers in the plasma of both treatments did not indicate any possible neuronal injury or dysfunction at baseline. In comparison to normal sociodemographic ranges, the expressions were as follows: (1) normal: NfL, DA, PEDF; (2) elevated: BDNF, CC4, cystatin c and (3) low: pre-albumin, RANTES, ApoC3, AGP, A1AT and ICAM-1.

### Blood CD8 + T lymphocytes (CD8 + HLADR + CD38 +) activation

In week 24, the placebo arm median (IQR) was 4.2 (3–6.1) % compared with the lithium arm median (IQR) of 4.2 (3–6.1) %, *p* = 0.54. Treatment exposure did not result in significant changes in either treatment arm (Supplementary Table 1).

### Plasma dopamine

**Fig. 1:**
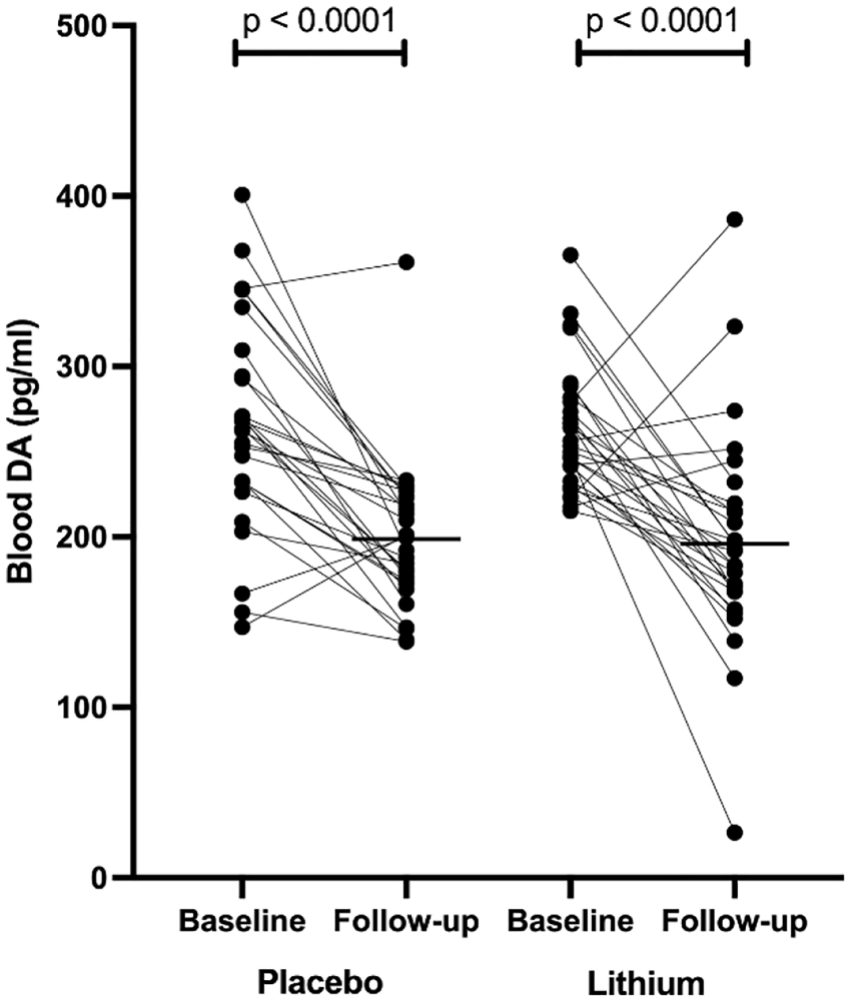
Changes in plasma or blood DA after 24 weeks of exposure. Baseline indicates before the intervention, and follow-up indicates after the intervention (week 24). Statistically significant changes are seen in both treatment arms (Mann–Whitney *U*-test for paired analysis of the nonparametric distribution of values)

Both treatment arms showed statistically significant (*p* < 0.0001) changes in dopamine (DA) concentration at the end of the intervention (24 weeks). In both groups, the changes remained statistically significant (*p* < 0.002) after post hoc FDR (Fig. 1, Supplementary Table 1). In the placebo group, the median (IQR) dopamine concentration was reduced from the median (IQR) of 262.3 (231.4–301.6) to 199.5 (173.3–225.4) pg/ml, a median difference of − 62.8 pg/ml. In the lithium group, the median (IQR) DA concentration was reduced from 249.9 (229.5–279.2) to 191.8 (168.0–217.5) pg/ml, a median difference of − 58.1 pg/ml.

### Plasma BDNF

A nearly significant (*p* = 0.05) change in the lithium group was observed in the concentration of BDNF at the end of the intervention (Supplementary Table 1). The median (IQR) concentration of BDNF dropped from a median of 36.51 (27–54) ng/ml to 29.63 (22–52) ng/ml, a median difference of − 5.059 (− 15–4). Due to the lack of statistical significance, the false discovery rate (FDR) approach was not used in our analysis.

### CSF biomarkers

Biomarkers in the CSF of both treatments did not indicate any possible neuronal injury or dysfunction at baseline. In comparisons to normal sociodemographic ranges, the expressions were as follows: (1) normal: sAPPα, dopamine and BDNF; (2) elevated: sAPPβ and (3) low: NfL Aβ38, Aβ40, Aβ42.

The concentrations of sAPPα (*p* = 0.01; adjusted *p* = 0.05) and sAPPβ (*p* = 0.05, adjusted *p* = 0.05) differed significantly between the two arms at 24 weeks. The sAPPα and sAPPβ were higher in the placebo group with median differences of 174.5 pg/ml, and 380.5 pg/ml, respectively. There was a significant change in the sAPPα concentration in the placebo arm, with a median increase of 38.50 (25–53) pg/ml and *p* = 0.03. Post hoc FDR correction removed this effect with adjusted *p* = 0.15 (Supplementary Table 3). No changes in the concentration of sAPPα were observed in the lithium arm.

## Discussion

The present study presents a secondary analysis of a large dataset of biomarkers analyzed from the blood/plasma and CSF during a 24-week randomized placebo-controlled clinical trial of patients with HAND. We conducted multiple biomarker comparisons, some of which are validated as biomarkers of HAND and others from Alzheimer’s dementia. We hypothesized that the AD biomarkers could help determine the effects of neuroprotective agents because of the similarities in the pathogenesis of HAND and AD. Plasma DA was significantly changed following treatment with lithium. The reduction in the plasma DA was also reduced in the placebo arm with a similar statistical significance of *p* = 0.002.

These findings could not support the neuroprotective benefits of lithium in moderate to severe HAND. Contrary, a previous study showed some evidence of lithium neuroprotection against HIV. In a small pilot study of PWH with HAND, lithium treatment for 10 weeks normalized neuronal metabolism and integrity (Schifitto et al. 2009). Evidence of neuroprotection was determined as an increase in neuronal glutamate/glutamine peak, an increase in white matter fractional anisotropy, and a reduction in mean diffusivity. There is also evidence that lithium treatment reverses HIV-related injuries in animal studies. Dou et al. (2005) found that lithium restored microtubule-associated protein 2 neurites and synaptic density in murine HIV encephalitis models (Dou et al. 2005). In addition, lithium reduced the activity of GSK-3-β. In another laboratory experiment, Tat infection of murine neurons resulted in a GSK-3-β activity increase. However, lithium treatment of the infected neurons resulted in attenuating the GSK-3-β activity (Maggirwar et al. 1999). Lithium is also well known for its ability to promote BDNF production (Yasuda et al. 2009).

It is also relevant to note that despite the attempt to use a broader approach to neuropathogenesis, the selected biomarkers are not directly linked with the putative pathway of lithium mechanism of action except for BDNF. A reduction in the BDNF occurs in various neuropsychiatric disorders and neurodegenerative disorders (Benussi et al. 2017). Changes in BDNF levels are also apparent in the blood of subjects with CNS diseases (Ventriglia et al. 2013). The plasma and CSF levels of BDNF have been used as markers to show cognitive status, and a correlation between plasma BDNF and brain levels has been suggested in animal and human studies (Balietti et al. 2018). In accordance, less BDNF was found in the brains of HIV-infected persons with HIV dementia than those without, possibly due to an impaired ratio of proBDNF to mature BDNF (Bachis et al. 2012).

Lithium response is associated with the Val66Met functional polymorphism of the BDNF gene located on chromosome 11p13 (Rybakowski 2014; Dmitrzak-Weglarz et al. 2008), and it was suggested that the therapeutic effects of lithium might be in part via modulation of BDNF (Castrén and Kojima 2016). However, this was not confirmed in other populations except Caucasians (Michelon et al. 2006). In our study, BDNF levels were reduced in the plasma of HIV-infected individuals on ART following lithium treatment, indicating a possible cognitive decline in the future in these individuals. The reasons for reduced serum BDNF levels in individuals receiving lithium remain unknown. It is possible that our patients were inadequate lithium responders as lithium did not recover GDS, and it is known that excellent lithium responders are associated with normal blood BDNF levels (Rybakowski 2014). In another study with euthymic adolescents with bipolar disorder, a lower BDNF level was detected in their blood after taking lithium (Cevher et al. 2016). The variations in the BDNF gene promoter region affect the expression of BDNF and its role in various neuropsychiatric disorders. For example, in an experiment with mice neurons, the antimanic effects of lithium were linked with BDNF modulation, which was not the case with the antidepressant effects (Gideons et al. 2017). These findings suggest that lithium’s action can be influenced not only by the neuropsychiatric status of patients but also by variations in the BDNF gene’s promoter region which affects the expression of BDNF (Hing et al. 2012). BDNF was included in our analysis, but no changes were observed following treatment with lithium.

Elevated DA results in activation of GSK-3-β, and lithium antagonizes this effect due to inhibition of GSK-3-β (Beaulieu et al. 2004). Lithium has been shown to regulate altered DA function (Malhi and Outhred 2016). HIV infection is associated with increased DA concentrations in CSF (Scheller et al. 2010). In PWH with the DAT10/10-repeat allele (Horn et al. 2013), we expected to find reduced DA levels. In our study, we could not see an effect of lithium on CSF DA concentrations due to the small number of patients with available CSF, and we did not check for polymorphisms in our population. However, after the intervention, we found a statistically significant reduction in plasma DA in both arms. DA in plasma is classified as a hormone rather than a transmitter, and three peripheral systems modulate peripheral catecholamines, including DA: the sympathetic branch of the autonomous nervous system, the autocrine/paracrine DA system and the adrenomedullary hormonal system, producing large amounts of catecholamines in response to acute stress or elevated arousal (Laverty 1978; Tank and Wong 2015). In our study, both treatment arms showed significant changes in DA levels after the intervention, suggesting that the observed DA reduction is independent of lithium pharmacotherapy. We can postulate that this reduction in DA concentration is because participants were more nervous during the examination at the beginning of the study (baseline) than at the follow-up visit. Thus, what we see in our study is likely a relative reduction in plasma DA level due to the elevated DA level at baseline caused by stress and anxiety of the anticipated clinical examination of the participants. Nervousness is associated with increased epinephrine which is approximately equivalent to that of dopamine concentrations in the plasma (Van Loon 1983). The observed decrease in dopamine concentrations in both treatment arms may therefore only reflect a normalization of dopamine concentrations that were elevated at baseline due to nervousness.

Unfortunately, we do not have serial blood pressure measurements to support our conjecture and the only study we know of that assessed a link between plasma catecholamine levels and anxiety and found no statistically significant changes used visual anxiety stressors (Gutierrrez-Martin et al. 2022) and not acute passive intrinsic stress as we might have in our study.

Because of the similarities between HAND and AD pathophysiology, we explored possible changes in the AD biomarkers at the end of the intervention (24 weeks). However, lithium did not change any of these biomarkers. In experimental animal models of AD, lithium's ability to prevent or reduce AD pathology has been demonstrated. Lithium was shown to reduce amyloid-beta synthesis in drosophila models of AD (Sofola-Adesakin et al. 2014). In a traumatic brain injury mouse model, lithium was also shown to reduce the synthesis of amyloid-beta and Tau protein phosphorylation (Yu et al. 2012). There is a possibility that the lack of lithium evidence of neuroprotection may be due to some factors that distinguish AD from HAND. The AD biomarkers of disease progression came from an older population than the cohort (Hye et al. 2014). The means (SD) age of participants in our study was 7.8 years younger than that of Hye et al. (2014), (mean (SD) 39 years vs. 76 years). While Hye et al. (2014) biomarkers demonstrated high sensitivity and specificity for predicting progress from MCI to AD, HAND has been described as a stable neurocognitive disorder during viral suppression (Sacktor et al. 2016). Furthermore, if a severe form of neuropathology is present in patients with moderate to severe HAND, the utility of these biomarkers may be compromised.

It is important to note that some of the biomarkers were within normal ranges, indicating no evidence of ongoing injury or neuronal dysfunction in both treatment arms. In addition, participants showed improvement in the GDS, likely secondary to the practice effect (Decloedt et al. 2016). One of the robust reasons for the HAND in this study that would not be mitigated by lithium is the legacy effect (Nightingale et al. 2014). Therefore, in this study, the presence of HAND was not necessarily indicative of persistent viral neuropathogenesis. Furthermore, the inclusion criteria included viral suppression and adequate ART. Even though CSF viral replication can continue even when there is evidence of viral suppression in the plasma, participants with CSF viral escape would be expected to demonstrate progressive neuropathology, which should manifest with clinical signs. In this study, participants did not show evidence of progressive neurological fallout (Decloedt et al. 2016).

In our study, over 80% of the participants were treated with non-nucleoside reverse transcriptase inhibitor-based ART regimens, mainly efavirenz (EFV). In vitro studies found that EFV and specifically its metabolite 8-hydroxy-EFV (8-OH-EFV) may cause neurotoxicity at therapeutic concentrations (Robertson et al. 2012). Although clinical data to support EFV neurotoxicity is conflicting and sparse, neurocognitive performance improved in participants (PWH) upon stopping EFV (Robertson et al. 2010). This could imply that the neurotoxic effects of EFV or 8-OH-EFV could confound the neuroprotection conferred by lithium treatment. However, our group found no association between CSF EFV or 8-OH-EFV concentrations and cognitive impairment in a previous investigation that included participants from this cohort. (Decloedt et al. 2019).

## Limitations

The study results should be interpreted considering various limitations. The participants in our study are homogeneous in terms of ethnicity and gender. Participants are mainly middle-aged females. We also analyzed many biomarkers from a small study sample size which may result in false-positive findings. Despite improving the duration of treatment compared with previous studies, we cannot rule out that prolonged exposure to lithium may cause some changes in the expression of biomarkers. Moreover, most of all, the expression of the biomarkers in both treatment groups indicated no active neuronal injury or dysfunction before the interventions.

## Conclusions

There was no evidence of lithium neuroprotection through surrogate biomarkers in this study. At baseline, neither plasma nor CSF concentrations indicated neuronal injury, which may explain the negative findings. This is, therefore, a potential confounder for this study. Future studies with participants with evidence of ongoing neuronal injury should be conducted to determine whether lithium provides neuroprotection.

## Supplementary Information

Below is the link to the electronic supplementary material.

Supplementary file1 (DOCX 41 KB)

## Data Availability

Data will be made available on request.
