# Supplementary material for: Blood and cerebrospinal fluid biomarker changes in patients with HIV-associated neurocognitive impairment treated with lithium: analysis from a randomised placebo-controlled trial
Source: J Neurovirol. 2023 Feb 15;29(2):156–66. doi: 10.1007/s13365-023-01116-4 (PMC10185609; doi:10.1007/s13365-023-01116-4)
Supplement: Supplementary file 1 — Supplementary file1 (DOCX 41 KB) [file 13365_2023_1116_MOESM1_ESM.docx]

Supplementary tables

**Table 1 The magnitude of change in HAND plasma/blood biomarkers after intervention**

|  | **Placebo arm** | | | | **Lithium** | | | |
| --- | --- | --- | --- | --- | --- | --- | --- | --- |
| **Biomarker** | **Week 0** | **Week 24** | **Change** | **P-value** | **Week 0** | **Week 24** | **Change** | **P-value** |
| **^a^CD8+ Activation (%)** |  |  |  |  |  |  |  |  |
| Number | 26 | 26 |  |  | 24 | 24 |  |  |
| Mean (SD) | 4.7 (3.5) | 5.1 (3.6) | 0.34 (3.1) |  | 6.5 (6.1) | 6.1 (5.1) | -0.37 (4.9) |  |
| Median (IQR) | 4.1 (2.1 – 5.5) | 4.2 (3 – 6.1) | 0.53 (-1 – 2.1) | 0.30 | 4.9 (2.8 – 7) | 4.7 (2.7 – 8.7) | -0.69 (-2.1 – 2.3) | 0.88 |
| 95%CI | 3.3 – 6.2 | 3.62 – 6.5 | -0.9 – 1.6 |  | 3.9 – 9 | 3.9 – 8.3 | -2.4 – 1.7 |  |
| **^a^DA (pg/ml)** |  |  |  |  |  |  |  |  |
| Number | 28 | 28 |  |  | 29 | 29 |  |  |
| Mean (SD) | 266.9 (62.24) | 198.8 (43.57) | -68.11(59.07) | <0.0001^*^ | 262.7 (36.75) | 196.0 (63.85) | -66.68 (73.64) | <0.0001^*^ |
| Median (IQR) | 262.3 (231.4 – 301.6) | 199.5 (173.3 – 225.4) | -62.8 | 0.002^#^ | 249.9 (229.5 – 279.2) | 191.8 (168.0 – 217.5) | --58.1 | 0.002^#^ |
| 95% CI | 242.8 – 291.0 | 181.9 – 215.7 | -91.02 – -45.2 |  | 248.7 – 276.7 | 171.8 – 220.3 | -94.69 – -38.67 |  |
| **^a^BDNF (ng/ml)** |  |  |  |  |  |  |  |  |
| Number | 28 | 28 |  |  | 28 | 28 | 28 |  |
| Mean (SD) | 37.73 (27.16) | 36.66 (25.47) | -1.062 (21.88) | 0.49 | 40.26 (20.27) | 34.96 (19.14) | -5.306 (14.80) | 0.05 |
| Median (IQR) | 35.6 (22 – 46) | 28.6 (19.2 – 50) | -4.809 (-20 - 11) |  | 36.51 (27 – 54) | 29.63 (22 – 52) | -5.059 (-15 – 4) |  |
| 95% CI | 27.20 – 48.26 | 26.79 – 46.54 | -9.547 – 7.423 |  | 32.40 – 48.12 | 27.54 – 42.38 | -11.04 – 0.43 |  |
| **^a^NfL (pg/ml)** |  |  |  |  |  |  |  |  |
| Number | 30 | 30 |  |  | 29 | 29 |  |  |
| Mean (SD) | 6.07 (2.90) | 6.72 (4.67) | 0.64 (2.97) |  | 5.38 (2.31) | 5.52 (2.97) | 0.14 (2.56) |  |
| Median (IQR) | 5 (4.4 – 7.8) | 5.02 (4 – 7.6) | -0.14 (-0.97 – 1.8) | 0.78 | 4.91 (3.6 – 6.5) | 4.78 (3.9 – 6.3) | -0.18 (-0.9 – 0.33) | 0.37 |
| 95%CI | 4.99 – 7.16 | 4.97 – 8.46 | -0.74 - 1.11 |  | 4.50 – 6.26 | 4.39 – 6.65 | -0.68 – 0.31 |  |

^a^Wilcoxon test, ^*^ statistically significant, ^#^adjusted p-value

HAND: HIV-associated neurocognitive disorder, NfL: Neurofilament light chains, DA: Dopamine, SD: Standard deviation, IQR: Interquartile range

**Table 2 The magnitude of change in AD plasma/blood biomarkers after intervention**

|  | **Placebo arm** | | | | **Lithium arm** | | | |
| --- | --- | --- | --- | --- | --- | --- | --- | --- |
| **Biomarker** | **Week 0** | **Week 24** | **Differences** | **P-value** | **Week 0** | **Week 24** | **Differences** | **P-value** |
| **^a^ApoC3 (mcg/ml)** |  |  |  |  |  |  |  |  |
| **Number** | 30 | 30 |  |  | 29 | 29 |  |  |
| Mean (SD) | 3.91 (2.22) | 3.95 (2.19) | 0.042 (2.541) | 0.74 | 3.87 (1.63) | 4.25 (2.02) | 0.38 (2.19) | 0.49 |
| Median (IQR) | 3.17 (2.39 – 4.49) | 3.34 (2.8 – 4.26) | 0.040 (-0.62 – 1.05) |  | 3.49 (2.74 – 4.9) | 3.68 (2.98 – 4.8) | -0.01 (-0.7 – 1.3) |  |
| 95%CI | 3.08 – 7.74 | 3.136 – 4.770 | -0.905 – 0.992 |  | 3.25 – 4.49 | 3.48 – 5.01 | -0.45 – 1.21 |  |
| **Pre-Albumin^1^ (mg/dL)** |  |  |  |  |  |  |  |  |
| Number | 30 | 30 |  |  | 29 | 29 |  |  |
| Mean (SD) | 6.59 (2.9) | 7 (2.98) | 0.41 (3.1) | 0.45 | 6.67 (3.35) | 7.34 (3.64) | 0.67 (2.79) | 0.45 |
| Median (IQR) | 6.18 (4.5 – 7.8) | 6.64 (4.9 – 8.4) | 0.9 (-1.6 – 2.3) |  | 5.78 (4.32 – 9) | 6.13 (5 – 9) | 0.47 (-1.4 – 2.3) |  |
| 95% CI | 5.5 – 7.7 | 5.9 – 8.1 | -0.75 – 1.59 |  | 5.39 – 7.95 | 5.96 – 8.73 | -0.39 – 1.73 |  |
| **AGP^1^ (mcg/ml)** |  |  |  |  |  |  |  |  |
| Number | 27 | 27 |  |  | 24 | 24 |  |  |
| Mean (SD) | 263.4 (161.9) | 225.5 (138.3) | -37.88 (196.20) | 0.22 | 214.6 (171.60) | 208.1 (113.5) | -6.52 (188.1) | 0.68 |
| Median (IQR) | 229.5 (165.2 – 333.5) | 179.5 (140.9 – 297.9) | -53.83 (-161.9 – 40.61) |  | 183 (137 – 269) | 207 (125 – 261) | -15.19 (-93 – 74) |  |
| 95% CI | 199.3 – 327.5 | 170.8 – 280.20 | -115.50 – 39.73 |  | 142.1 – 287.10 | 160.2 – 256.0 | -85.95 – 72.91 |  |
| **^a^A1AT (mcg/ml)** |  |  |  |  |  |  |  |  |
| Number | 27 | 27 |  |  | 24^2^ | 24 |  |  |
| Mean (SD) | 48.89 (5.729) | 56.69 (24.36) | 8.096 (25.70) | 0.43 | 48.66 (6.33) | 49.29 (6.68) | 0.64 (8.35) | 0.71 |
| Median (IQR) | 49.08 (44.85 – 53.22) | 48.92 (46.84 – 58.02) | -2.00 (-4.59 – 13.56) |  | 48.07 (45 – 54) | 49.77 (45 – 54) | -2.02 (-4.1 – 9) |  |
| 95% CI | 46.33 – 50.86 | 47.05 – 66.33 | -2.072 – 18.26 |  | 45.98 – 51.33 | 46.48 – 52.11 | -2.89 – 4.16 |  |
| **^a^PEDF (mcg/ml)** |  |  |  |  |  |  |  |  |
| Number | 30 | 30 |  |  | 28 | 28 |  |  |
| Mean (SD) | 5.19 (1.52) | 6.72 (6.17) | 1.52 (6.65) | 0.66 | 5.49 (1.65) | 5.22 (0.95) | -0.28 (1.65) | 0.67 |
| Median (IQR) | 5.22 (4.13 -6.21) | 5.17 (4.21 – 6.94) | 0.16 (-1.02 -1.55) |  | 5.09 (4.3 – 6.2) | 5.26 (4.4 – 5.6) | -0.27 (-1 – 0.7) |  |
| 95% CI | 4.63 – 5.77 | 4.42 – 9.03 | -0.96 – 4.00 |  | 4.86 – 6.14 | 4.85 – 5.59 | -0.92 – 0.36 |  |
| **^a^CC4 (mcg/ml)** |  |  |  |  |  |  |  |  |
| Number | 28 | 28 |  | 0.43 | 27 | 27 |  | 0.56 |
| Mean (SD) | 138.4 (28.22) | 150.6 (49.98) | 12.14 (51.38) |  | 160.8 (44.99) | 157.3 (35.81) | -3.59 (41.60) |  |
| Median (IQR) | 142.6 (109.9 – 156.2) | 140.7 (113.3 –167.3) | 1.76 ( -8.29 –17.61) |  | 151 (124 – 190) | 159 (133 – 175) | -3.020(-13 – 5) |  |
| 95% CI | 127.5 – 149.4 | 131.2 – 169.9 | -7.778 – 32.07 |  | 143.11 – 178.6 | 143.1 – 171.4 | -20.05 – 12.86 |  |
| **^b^ICAM-1 (ng/ml)** |  |  |  |  |  |  |  |  |
| Number | 30 | 30 |  |  | 29 | 29 |  |  |
| Mean (SD) | 1967 (736.4) | 1868 (656.1) | -98.29 (1005) | 0.59 | 2105 (1111) | 2055 (1695) | -49.76 (1194) | 0.62 |
| Median (IQR) | 2188 (1365 – 2439) | 1782 (1448 – 2231) | -331.9 (-969 –982) |  | 1985 (1383 – 2542) | 1907 (1176 – 2395) | -15.13 (-852 – 282) |  |
| 95% CI | 1668 – 2242 | 1623 – 2114 | -473.7 – 277.1 |  | 1682 – 2525 | 1410 – 2700 | -503.2 – 404.3 |  |
| **^a^RANTES (ng/ml)** |  |  |  |  |  |  |  |  |
| Number | 30 | 30 |  |  | 29 | 29 |  |  |
| Mean (SD) | 474.1 (182.5) | 471.6 (417.3) | -2.480 (371.7) | 0.12 | 481.1 (258.1) | 488.2 (252.6) | 7.14 (305.9) | 0.70 |
| Median (IQR) | 441.3 (340.5 – 581.9) | 378.8 (283 – 506.0) | -9.9(-167– 68.44) |  | 429(340 – 572) | 439 (360 – 551) | -6.7 (-151 – 214) |  |
| 95% CI | 405.91 – 542.2 | 315.8 – 627.4 | -141.3 – 136.3 |  | 382.9 – 579.3 | 392.2 – 584.3 | -109.2 – 123.5 |  |
| ^b^Clusterin (mcg/ml) |  |  |  |  |  |  |  |  |
| Number | 30 | 30 |  |  | 29 | 29 |  |  |
| Mean (SD) | 1233 (288.8) | 1179 (345.8) | -53.94 (410.5) | 0.48 | 1289 (379.7) | 1143 (270.8) | -145.9 (436.2) | 0.08 |
| Median (IQR) | 1245 (1065 – 1423) | 1168 (876 –1559) | -87.88 ( -281.9 – 206.6) |  | 1308 (968 – 1565) | 1118 (919 – 1292) | -91.3 (-485 – 191) |  |
| 95% CI | 1125 – 1341 | 1050 – 1308 ^2^ | -207.2 – 99.35 |  | 1145 - 1433 | 1040 - 1246 | -311.8 – 20.01 |  |
| **^c^Cystatin c (mcg/ml)** |  |  |  |  |  |  |  |  |
| Number | 8 | 6 |  |  | 7 | 3 |  |  |
| Mean (SD) | 1.23 (0.15) | 1.28 (0.17) | ----- | 0.38 | 1.40 (0.27) | 1.33 (0.16) | ------ | 0.83 |
| Median (IQR) | 1.18 (1.13 – 1.38) | 1.24 (1.18 – 1.38) |  |  | 1.35 (1.2 – 1.6) | 1.38 (1.15 – 1.5) |  |  |
| 95% CI | 1.35 – 1.35 | 1.45 – 1.45 |  |  | 1.16 – 1.65 | 0.93 – 1.73 |  |  |

^1^Wilcoxon test, ^b^Parametric paired t-test, ^c^Unpaired Man Whitney Test, ^*^ statistically significant. AD: Alzheimer’s dementia

**Table 3 The magnitude of change in the CSF biomarkers after intervention**

|  | **Placebo arm** | | | | **Lithium arm** | | | |
| --- | --- | --- | --- | --- | --- | --- | --- | --- |
| **Biomarker** | **Week 0** | **Week 24** | **Difference** | **P-value** | **Week 0** | **Week 24** | **Difference** | **P-value** |
| **^b^DA (ng/ml)** |  |  |  |  |  |  |  |  |
| Number | 4 | 4 |  |  | 2 | 2 |  |  |
| Mean (SD) | 1.95 (1.06) | 1.18 (0.61) | -0.77 (1.04) |  | 2.90 (3.18) | 0.95 (0.89) |  |  |
| Median (IQR) | 2.28 (0.8 – 2.7) | 0.97 (0.8 – 1.8) | -0.89 (-1.7 – 0.28) | 0.38 | 2.90 (0.65 – 5.2) | 0.95 (0.32 – 1.58) |  | Few pairs |
| 95% CI | 0.27 – 3.63 | 0.21 – 2.15 | -2.43 – 0.89 |  | -25.67 – 31.48 | -7.02 – 8.91 |  |  |
| **^b^BDNF (ng/ml)** |  |  |  |  |  |  |  |  |
| Number | 5 | 5 |  |  | 4 | 4 |  |  |
| Mean (SD) | 0.93 (0.65) | 1.52 (0.65) | 0.59 (0.75) |  | 1.41 (0.48) | 1.18 (0.28) | -0.22 (0.35) |  |
| Median (IQR) | 0.54 (0.44 – 1.6) | 1.31 (1 – 2.2) | 0.62 (-0.06 – 1.2) | 0.19 | 1.424 (0.94 – 1.85) | 1.23 (0.89 – 1.42) | -0.29 (-0.51 – 0.13) | 0.38 |
| 95% CI | 0.12 – 1.73 | 0.72 – 2.33 | -0.33 – 1.53 |  | 0.642 – 2.17 | 0.739 – 1.63 | -0.79 – 0.34 |  |
| **^b^NfL (pg/ml**) |  |  |  |  |  |  |  |  |
| Number | 6 | 6 |  |  | 6^1^ | 6 |  |  |
| Mean (SD) | 384.0 (88.69) | 392.3 (124.0) | 8.333 (149.2) | >0.99 | 362.8 (177.0) | 338.7 (178.1) | -24.17 (40.44) |  |
| Median (IQR) | 361.5 (306 – 480) | 341.0 (303 – 535) | -8.50 (-90.5 – 103) |  | 352.0 (206 – 519) | 302.5 (182 – 516) | -11.50 (-51 – 3) | 0.20 |
| 95% CI | 290.9 – 477.1 | 262.2 – 522.4 | -148.3 – 164.9 |  | 177 – 548.6 | 151.8 – 525.6 | -66.61 – 18.28 |  |
| **^b^Aβ38 (pg/ml)** |  |  |  |  |  |  |  |  |
| Number | 6 | 6 |  |  | 6^a^ | 6 |  |  |
| Mean (SD) | 1794 (538.5) | 1873 (464.1) | 79.33 (392.6) | 0.56 | 1126 (570.1) | 1125 (486.1) | -0.833 (146.8) |  |
| Median (IQR) | 2036 (1302 – 2186) | 1986 (1441 – 2221) | 235.0 (-223 – 324) |  | 1045 (615 – 1722) | 999.0 (659 – 1717) | -0.5 (-130 - 108) | 0.99 |
| 95% CI | 1229 – 2359 | 1386 – 2360 | -332.7 – 491.3 |  | 527.9 – 1724 | 615.2 – 1635 | -154.9 – 153.2 |  |
| **^a^Aβ40 (pg/ml)** |  |  |  |  |  |  |  |  |
| Number | 6 | 6 |  |  | 6 | 6 |  |  |
| Mean (SD) | 5055 (1569) | 4919 (1296) | -136.2 (145) |  | 3134 (1748) | 3114 (1374) | -19.33 (470.3) |  |
| Median (IQR) | 5199 (3754 – 6357) | 4965 (3768 – 6152) | 359.0 (-834 – 751) | 0.83 | 3016 (1544 – 4591) | 2803 (1905 – 4527) | -20.00 (-354 – 361) | 0.92 |
| 95% CI | 3408 – 6701 | 3558 – 6279 | -1659 – 1389 |  | 1299 – 4968 | 1672 – 4556 | -512.9 – 474.2 |  |
| **^a^Aβ42 (pg/ml)** |  |  |  |  |  |  |  |  |
| Number | 6 | 6 |  |  | 6 | 6 |  |  |
| Mean (SD) | 456.3 (167.4) | 418.8 (112) | -37.50 (151.2) |  | 260.7 (161.7) | 267.5 (118.8) | 6.833 (50.69) |  |
| Median (IQR) | 468.5 (331 - 602) | 395.5 (353 – 510) | 28.50 (-212 – 76) | 0.57 | 248.0 (114 – 396) | 251.0 (160 – 375) | 15.50 (-41 – 46) | 0.76 |
| 95% CI | 280.6 – 632.0 | 301.7 – 535.9 | -196.2 – 121.2 |  | 91.02 – 430.3 | 142.8 – 392.2 | -46.37 – 60.03 |  |
| **^b^sAPPα (pg/ml)** |  |  |  |  |  |  |  |  |
| Number | 6 | 6 |  |  | 6 | 6 |  |  |
| Mean (SD) | 225.8 (63.65) | 265.8 (75.13) | 40.00 (21.79) | 0.03^*^ | 114.7 (63.96) | 119.2 (72.05) | 4.500 (16.24) | 0.56 |
| Median (IQR) | 247.5 (177 – 270) | 298.5 (193 – 323) | 38.50 (25 – 53) | 0.15^#^ | 81.50 (71 – 191) | 81.00 (68 – 211) | 8.5(-11 – 16) |  |
| 95% CI | 159.0 – 292.6 | 187.0 – 344.7 | 17.13 – 62.87 |  | 47.55 – 181.8 | 43.56 – 194.8 | -12.55 – 21.55 |  |
| **^b^sAPPβ (pg/ml)** |  |  |  |  |  |  |  |  |
| Number | 6 | 6 |  |  | 6 | 6 |  |  |
| Mean (SD) | 527.0 (152.1) | 607.7 (192.5) | 80.67 (92.82) |  | 400.2 (297.9) | 298.5 (157.8) | -101.7 (291.3) |  |
| Median (IQR) | 526.5 (406 – 686) | 700 (371 – 739) | 84.5(9 – 163) | 0.16 | 320.0 (182 – 603) | 235.5 (165 – 487) | 18.50 (-202 – 37) | 0.43 |
| 95% Cl | 367.4 – 686.6 | 405.6 – 809.7 | -16.74 – 178.1 |  | 87.59 – 712.7 | 132.9 – 464.1 | -407.4 – 204.0 |  |

^a^Paired student-test,^b^Wilcoxon, ^*^ statistically significant, ^#^adjusted p value

NfL: Neurofilament light chains, BDNF: Brain-derived neurotrophic factors, DA: Dopamine, SD: Standard deviations, IQR: Interquartile range, LP: lumbar puncture
